# Impact of an antimicrobial time-out program on antimicrobial consumption rate in hospitalized patients: a quasi-experimental study on the national antimicrobial stewardship program in Iran: Iranian antimicrobial stewardship program

**DOI:** 10.1186/s40780-025-00451-4

**Published:** 2025-05-20

**Authors:** Mohammadreza Salehi, Marzieh Arabi, Hossein Khalili, Yunes Panahi, Erta Rajabi, Esmaeil Mohammadnejad, Amir-mohammad Yaryari, Arash Seifi, Mitra Barati, Kousha Farhadi

**Affiliations:** 1 Research Center for Antibiotic Stewardship & Antimicrobial Resistance, Department of Infectious Diseases, Imam Khomeini Hospital Complex, Tehran University of Medical Sciences, Tehran, Iran; 2 Department of Infectious Diseases, School of Medicine, Imam Khomeini Hospital Complex, Tehran University of Medical Sciences, Tehran, Iran; 3 Department of Clinical Pharmacy, School of Pharmacy, Tehran University of Medical Sciences, Tehran, Iran; 4 Pharmacotherapy Department, Faculty of Pharmacy, Baqiyatallah University of Medical Sciences, Tehran, Iran; 5 Department of Medical-Surgical Nursing and Basic Sciences, School of Nursing & Midwifery, Tehran University of Medical Sciences, Tehran, Iran; 6 Paediatric Infectious Diseases Research Centre, Iran University of Medical Science, Tehran, Iran

**Keywords:** Antimicrobial stewardship, Antibiotic consumption, Antimicrobial time-out, Hospitals, Iran, Therapeutic use

## Abstract

**Background:**

This study evaluated the impact of the national antimicrobial stewardship program (NASP) on the consumption of antimicrobial agents.

**Methods:**

A quasi-experimental study was conducted on hospitalized patients at a referral hospital in Tehran, Iran. We compared the antimicrobial-defined daily dose (DDD) and antimicrobial consumption index (ACI) between the third quarter of 2022 (before the implementation of NASP after the COVID-19 pandemic in October 2022) and the same timeframe in 2023, following the NASP implementation. The NASP was based on antimicrobial time-out assessment. Within 72 h of prescribing meropenem, imipenem, linezolid, vancomycin, voriconazole, caspofungin, and amphotericin B liposomal, infectious disease specialists audited the clinical and microbiological evidence of patients to assess whether it was consistent with the correct prescription.

**Results:**

The antimicrobial consumption rate was assessed in 13,794 and 15,030 hospitalized patients during the third quarter of 2022 and the third quarter of 2023, respectively. The mean length of hospital stay and mortality rate showed no significant differences. The consumption of all restricted antimicrobials decreased. This reduction was significant for imipenem, caspofungin, vancomycin, and linezolid. The total cost of antimicrobial agents had a 22.24% reduction after the NASP implementation (*P* = 0.01).

**Conclusions:**

The antimicrobial time-out program was associated with a reduction in the use of antimicrobials, including imipenem, linezolid, and vancomycin and antifungals, such as caspofungin without increasing the length of stay and mortality rate. The NASP implementation can be recommended as a beneficial method for reducing the use of broad-spectrum antimicrobials.

## Background

Anti-microbial resistance (AMR) is a major global challenge emerged due to the increasing prevalence of inappropriate antimicrobial use [1]. This direct link between antimicrobial overuse and AMR has long been evident [2]. Antimicrobial are unique pharmaceutical agents whose effectiveness alters over time, and resistance to them can transfer to other bacteria through genetic modifications or phenotypic resistance [3, 4]. The careful use of antimicrobials by medical professionals can prevent bacterial resistance and other adverse effects of antimicrobials, such as infections caused by *Clostridioides (Clostridium) difficile* [5]. AMR could cause up to 10 million deaths per year by 2050, which would surpass heart disease and cancer as the leading causes of death. Its global costs are also estimated to exceed 100 trillion USD [6].

The increasing global burden of AMR and the associated costs and side effects pose a significant challenge to healthcare providers [7]. In order to address this issue, antimicrobial stewardship programs (ASP) have been developed and implemented in numerous nations [8–13]. Antimicrobial stewardship is a coordinated program designed to optimize the use of antimicrobial agents in order to manage AMR and improve patient outcomes [3]. Such programs involve monitoring and adjusting different aspects of antimicrobial therapy, including proper selection dose, duration, and method of administration [14]. Numerous studies have demonstrated the effectiveness of antimicrobial stewardship programs in reducing healthcare costs and antimicrobial resistance rates [8, 10–12].

In 2015, The World Health Organization (WHO) identified the Eastern Mediterranean region as one of the weakest regions in the world in the fight against AMR, possibly due to the indiscriminate administration of antimicrobials [15]. The results of an investigation on the national pharmaceutical wholesale data bank showed that the consumption of antimicrobials in Iran grew significantly between 2000 and 2016 [16]. Since 2016, the Iran’s Ministry of Health and Medical Education has implemented a mandatory national program in both inpatient and outpatient sections separately to monitor and control the excessive use of broad-spectrum antimicrobials prescription in all hospitals, healthcare centers, and insurance organizations throughout the country [17].

This study evaluated the effects of the national antimicrobial stewardship program (NASP) in a tertiary university hospital in Tehran, Iran.

## Methods

### National antimicrobial stewardship program (hospital section)

In early 2016, the instruction on the obligatory antmicrobial stewardship program in Iran was released by the Iran’s Ministry of Health and Medical Education in inpatient and outpatient sections separately to be implemented in all academic/public and private hospitals and healthcare centers and supervised by insurance organizations [17]. The instruction (hospital section) was based on an antimicrobial time-out program. Within 72 h of the initial prescription of meropenem, imipenem, linezolid, vancomycin, voriconazole, caspofungin, and amphotericin B liposomal, patients were reassessed by infectious disease specialists. If patients exhibited matching clinical symptoms, had risk factors for multi-drug resistant (MDR) pathogens, or showed microbiological or mycological evidence, the agent was continued. Finally, a clinical pharmacist reviewed and possibly adjusted the dosage, interactions, and duration of the antimicrobial agents approved for continuation. According to this protocol, piperacillin/tazobactam, aminoglycosides, quinolones, cephalosporins, and fluconazole were not restricted or monitored.

### Patients and settings

In a quasi-experimental study, we evaluated the antimicrobial consumption rate in hospitalized patients at a referral hospital in Tehran, Iran, from the first of July to the end of December 2022. This teaching hospital is equipped with 1400 beds across various surgical and internal medicine wards and with nearly 200 ICU beds. The medical staff consists of faculty members and specialized and sub-specialized assistants of the university. Medical orders, including antimicrobial regimens, are written by faculty members and specialized and subspecialized assistants. During the COVID-19 pandemic, the national antimicrobial stewardship program was affected significantly in hospitalized settings in Iran, similar to other countries, and was not implemented effectively, which resulted in an indiscriminate use of antimicrobials [18]. In mid-2022, due to a substantial decline in COVID-19 hospitalizations, the antimicrobial Stewardship Committee of the hospital mandated re-implementing the national antimicrobial stewardship guidelines. Starting on October 1st, 2022, these instructions were again in effect at the hospital. To eliminate the impact of the COVID-19 pandemic on patient hospitalization, we included data regarding only the three months before the implementation of NASP in October 2022. Therefore, we compared the antimicrobial consumption rate between the third quarter of 2022 and the same period in 2023, following the NASP implementation. The number of patients, bed-days, the type of antimicrobial agents administered, antimicrobial-defined daily dose (DDD), and DDD per 100 bed-days (antimicrobial consumption index, ACI) were recorded for each period, and the results were compared. The antimicrobial use in the study was derived from the DDD for each antimicrobial according to the ACI/DDD list of the WHO Collaborating Centre for Drug Statistics Methodology [19].

The study population included all hospitalized patients who were 18 years old or older. The exclusion criteria were patients with COVID-19, patients who died within 48 h of hospitalization, and patients who were discharged within the first 48 h of hospitalization.

The antimicrobials under study were categorized into two groups: restricted group (carbapenems (imipenem and meropenem), amphotericin B liposomal, vancomycin, linezolid, caspofungin, and voriconazole) and alternative group (cefepime, piperacillin/tazobactam, clindamycin, amikacin, gentamicin, fluconazole, ciprofloxacin, and levofloxacin).

### Data collection and analysis

The required data were obtained from the hospital’s pharmacovigilance department and the hospital information system. The extracted data were then compiled into an Excel spreadsheet, and the ACI attributed to each antimicrobial was calculated using the standard DDD defined by the WHO. Data were analyzed using the SPSS software (version 26.0, USA). Normality of data was analyzed using Kolmogorov-Smirnov test, then accordingly either the paired sample t-test or the Wilcoxon signed-rank test was performed. A *P*-value of < 0.05 was considered significant.

## Results

### Patients, hospitalization, and mortality

We assessed 13,794 and 15,030 patients during the third quarter of 2022 and third quarter of 2023, respectively, with an increase of 1236 (9%, *P* = 0.089) patients in 2023. The mean length of hospital stay (5.86 vs. 6.21 days) and mortality rate (2.49% vs. 2.52%) did not differ significantly (Table 1).

**Table 1:** The number of mean patients assessed, mean hospitalization time and mean mortality before and after NASP implementation

	Before	After	Change	Standard Deviation		*P* Value
				Before	After	
Mean monthly hospitalized patients	4598	5010	+412	264.35	178.37	0.89
Mean length of stay (days)	5.86	6.21	+ 0.35	1.25	0.24	0.66
Mean mortality rate	2.49%	2.52%	+ 0.02	6.25	14.57	0.264

### Restricted and alternative antimicrobial consumption

Following the implementation of the National Antimicrobial Stewardship Program (NASP), consumption rates of all restricted antimicrobials decreased numerically, with the total antibiotic consumption index (ACI) declining by 23.61% (*P* < 0.001). Meropenem exhibited the smallest reduction in ACI (-13.29%; *P* = 0.11), whereas imipenem showed the largest decrease (-64.96%; *P* < 0.001). For other primary antimicrobials, ACI reductions ranged between 20% and 30%. Among antifungals, only caspofungin demonstrated a statistically significant decline in ACI (-25.26%; *P* < 0.001). In contrast, the decreases for amphotericin B liposomal (-21.62%; *P* = 0.237) and voriconazole (-28.28%; *P* = 0.966) were not significant. Both vancomycin and linezolid showed moderate but significant reductions in ACI (-23.18%; *P* = 0.003 and − 25.06%; *P* = 0.026, respectively).

**Fig. 1:**
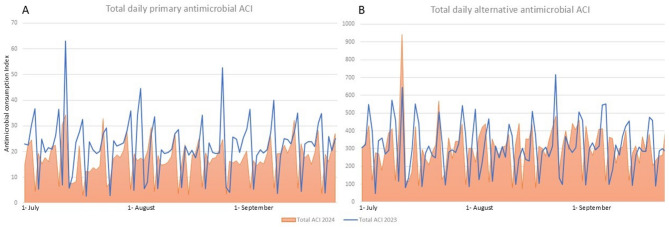
The comparison of total primary (**A**) and alternative (**B**) antimicrobial consumption rate before and after NASP implementation based on antimicrobial consumption index (ACI)

Among the assessed alternative antimicrobials, varying results were obtained. In case of fluoroquinolones, ciprofloxacin ACI decreased by 9.7% (*P* = 0.185), while levofloxacin ACI increased by 8.32% (*P* = 0.345). This disparity was also seen in aminoglycosides, with gentamicin showing a 4.78% decreased ACI and amikacin a 3.08% increased ACI (*P* = 0.469 and *P* = 0.524, respectively). Clindamycin’s ACI change was insignificant (+ 4.06%; *P* = 0.141). Notably, piperacillin-tazobactam (PTZ) exhibited a sharp increase in ACI (88.45%; *P* < 0.001), while cefepime (-49.22%; *P* < 0.001) and fluconazole (-62.52%; *P* < 0.001) showed marked declines. Unlike primary antimicrobials, the total ACI change for alternative antimicrobials remained nonsignificant (-9.66%; *P* = 0.175; Table 2; Fig. 1).

**Table 2:** The comparison of antimicrobial consumption rate in the restricted and alternative groups before and after NASP implementation based on defined daily dose and antimicrobial consumption index

Drug name	Defined Daily Dose (SD)		Mean Change (%)	*P* value	Antimicrobial Consumption Index (SD)		Mean Change (%)	*P* value
	Before	After			Before	After		
Restricted group								
^a^Amphotericin B liposomal	5.33 (6.0)	5 (7.91)	-18.29	^b^0.332	0.566 (0.624)	0.500 (0.624)	-21.62	^b^0.237
^a^Caspofungin	4.9 (4)	4 (3.4)	-22.51	^b^0.003	0.515 (0.418)	0.405 (0.341)	-25.26	^b^<0.001
^a^Linezolid	3.5 (3.5)	3 (2)	-21.84	^b^0.064	0.366 (0.378)	0.304 (0.198)	-25.06	^b^0.026
^a^Imipenem	21.875 (14.69)	7.25 (5.94)	-63.50	^b^<0.001	2.294 (1.542)	0.739 (0.604)	-64.96	^b^<0.001
^a^Meropenem	91.167 (39.667)	79.917 (40.125)	-9.88	^b^0.308	9.573 (4.074)	8.080 (4.128)	-13.29	^b^0.11
^a^Vancomycin	75.625 (32.88)	63.625 (24.56)	-20.14	^b^0.014	8.023 (3.365)	6.399 (2.535)	-23.18	^b^0.003
^a^Voriconazole	3 (4.9)	4 (6.5)	-25.47	^b^0.838	0.320 (0.522)	0.413 (0.643)	-28.28	^b^0.966
^c^Total	19405.02	15410.45	-20.58	^b^0.002	2040.70	1558.87	-23.61	^b^<0.001
Alternative group								
^a^Clindamycin	50.333(26.67)	56.5(32.729)	8.22	^b^0.059	5.344(2.793)	5.602(3.320)	4.06	^b^0.141
^a^Piperacillin/Tazobactam	24.549(13.902)	48.777(22.440)	95.92	^b^<0.001	2.568(1.413)	4.959(2.220)	88.45	^b^<0.001
^a^Amikacin	6.95(6.9)	8.00(7.90)	6.97	^b^0.315	0.733(0.712)	0.819(0.811)	3.080	^b^0.524
Gentamicin	23.441(9.863)	23.255(11.326)	-0.80	0.906	2.468(2.514)	2.350(1.142)	-4.78	0.469
^a^Cefepime	16.5(11.5)	11(11.625)	-34.36	^b^<0.001	2.134(1.500)	1.102(1.150)	-49.22	^b^<0.001
^a^Fluconazole	45.375(50.56)	14.5(31)	-60.89	^b^<0.001	4.765(5.312)	1.468(3.195)	-62.52	^b^<0.001
Ciprofloxacin	92.904(46.20)	87.078(44.51)	-6.27	0.398	9.765(4.844)	8.817(4.547)	-9.70	0.185
^a^Levofloxacin	30(22)	43.5(34.3)	37.78	^b^<0.001	3.746(2.770)	4.320(3.736)	8.32	^b^0.345
^c^Total	28727.79	27945.37	-2.72	^b^0.916	3132.37	2829.68	-9.66	^b^0.175

^a^_Median (Interquartile range),_^b^_Wilcoxon signed−rank test,_^c^_SUM_

### Financial burden

The evaluation of the total expenses associated with restricted and alternative antimicrobial use revealed a decrease of 22.64% and 12.54%, respectively (Table 3).

**Table 3:** Total money spent on restricted and alternative antimicrobials before and after NASP implementation

	Number of drugs		Before (USD)		After (USD)	Change (USD)	*P* value
Restricted antimicrobials		7	451017.32		350728.34	− 100288.98 (22.24%)	0.010
Alternative antimicrobials		8	170457.77	150271.49		–20186.28 (11.8%)	0.013

## Discussion

We observed a decline in the targeted antimicrobial consumption rate and costs after the antimicrobial time-out program implementation at a referral hospital in Tehran, Iran. This reduction did not have any negative impact on patient outcomes. Similarly, a comprehensive review of ASPs in Asia showed that they could effectively reduce the antimicrobial consumption rate without any effects on clinical outcomes [20].

In the antimicrobial time-out approach, to minimize inappropriate antimicrobial exposure, the antimicrobial regimen prescribed by the clinician is re-evaluated after or during a specified period of time (usually on the third day of treatment), when clinical improvement can be assessed and pathogens or disease-causing agents may have been identified [21]. In the method used in the NASP, an infectious disease specialist reviewed the clinical records, and the final assessment was made by a clinical pharmacist within the first three days of prescription. While previous studies have reported different impacts of antimicrobial time-out interventions on the antimicrobial consumption rate [21–23], our study showed that the time-out approach, as implemented in the NASP, could be effective in reducing the rate of antimicrobial consumption.

When comparing antibiotic consumption before NASP implementation, as reported in this study, with data from other Middle Eastern countries, notable differences emerge. For instance, a study in Oman involving hospitalized patients and audit-and-feedback interventions documented higher meropenem and piperacillin/tazobactam ACIs than those observed here. However, vancomycin prescription rates in the Omani study exceeded those in the current analysis [24]. Altaf et al. evaluated the efficacy of a culture-sensitivity, prescription-based ASP on antibiotic ACIs. The ACI for piperacillin/tazobactam, meropenem, and vancomycin were lower than the values found in this study. However, pre-ASP amikacin usage in their cohort was higher than the baseline rates reported here [25]. In contrast to Altaf et al.‘s findings, a Jordanian study implementing a comprehensive ASP found a piperacillin ACI of 8.2 per 1,000 patient bed-days, which is substantially higher than the 2.568 ACI observed in this study [26]. A Qatari pre-ASP study revealed lower ACIs for vancomycin, amikacin, ciprofloxacin, linezolid, and meropenem compared to this study’s baseline. However, levofloxacin and piperacillin/tazobactam ACIs in Qatar exceeded the pre-intervention rates reported here [27].

Pre-ASP antibiotic resistance patterns in Iran, as documented in prior literature, included methicillin-resistant *Staphylococcus aureus* (MRSA) in 77.20% of isolates, carbapenem-resistant Enterobacteriaceae (CRE) in 86.82%, *Klebsiella pneumoniae* carbapenemase (KPC) in 68.45%, imipenem/meropenem resistance in 8.90% of gram-negative bacteria, vancomycin-resistant Enterococcus (VRE) ranging from 4.80 to 55.54%, and meropenem-resistant *Pseudomonas aeruginosa* in up to 75% of cases [28–31]. Notably, compared to other Middle Eastern countries, Iran reported markedly higher frequencies of meropenem-resistant *P. aeruginosa*, CRE, MRSA, and KPC isolates [24, 32–35]. Additionally, cefepime consumption significantly decreased after NASP implementation, possibly due to optimized prescriptions after the reassessments by the infectious disease specialist or the clinical pharmacist due to inappropriate prescription, and an increased tendency to prescribe narrow-spectrum antibiotics [36, 37].

Carbapenems are considered the gold standard agents for the treatment of patients with extended spectrum beta-lactamase producing Enterobacterales (ESBL-PE) infections [38]. Studies have shown that carbapenem-sparing strategies can prevent the spread of MDR and extended drug resistance (XDR) Gram-negative microorganisms [39]. Similarly, our study suggests that the implementation of NASP could result in reduced consumption of carbapenems, specifically imipenem. The observed decrease in imipenem consumption might be linked to high baseline prescription rates of carbapenems as empirical therapy [40]. Furthermore, studies from Iranian teaching hospitals highlight that even when carbapenem use is clinically justified, the duration of therapy is often inappropriate. This mismanagement may inadvertently perpetuate carbapenem overuse [41–44] These findings align with Mousavi et al.’s report, which identified empirical imipenem prescribing without adequate therapeutic drug monitoring or adjustments based on culture results [45].

Tazobactam is a potent β-lactamase inhibitor, and piperacillin-tazobactam (PTZ) is a β-lactams/-lactamase inhibitor combination [46]. The results of a meta-analysis on studies comparing carbapenems with PTZ for the treatment of ESBL-PE infections showed that when PTZ was empirically prescribed in patients with suspected hospital-acquired infections, no significant difference in the mortality rate was observed [47]. The significant increase in PTZ consumption seen in our study is similar to findings of a study by Yeo et al., which reported a considerable increase in PTZ use after the implementation of the ASP [48]. Furthermore, similar trends have been documented in multiple studies [37, 49–51]. This shift could reflect an increased tendency to replace broad-spectrum antimicrobials following ASP implementation. Additionally, PTZ serves as a clinically alternative to carbapenems for treating infections caused by ESBL-PE [52, 53]; while also, PTZ is favored over carbapenems when resistance patterns allow, as it balances efficacy, resistance prevention, cost-effectiveness, while also posing a lower risk of *Clostridioides difficile* infection and microbiome disruption. Our study is also consistent with a similar study by Cook et al., in which the implementation of the ASP reduced the antimicrobial consumption by 27% [54]. In the same study, the use of antifungal drugs decreased by 28%, with fluconazole having the most significant reduction (70%), although not under the restriction group. They also reported a significant decrease in antimicrobial costs over five years, similar to our study [54]. Similar to our findings, Lo´pez-Medrano et al. reported a 20% reduction in caspofungin use after the ASP implementation [55].

Despite the observed decrease in ciprofloxacin use, levofloxacin experienced a sharper increase after NASP implementation, which could be due to targeted recommendations Although comprehensive national data on fluoroquinolone consumption are limited, studies estimate a 392% increase in fluoroquinolone use between 2000 and 2016 [56]. The high consumption of fluoroquinolones in Iran was restated in Nabovati et al.‘s study, where ciprofloxacin was among the most commonly prescribed antibiotics in 2018 [57]. esistance patterns vary by pathogen. For example, fluoroquinolone-resistant *Shigella* and *Salmonella* isolates in Iranian pediatric populations are relatively rare [58, 59], which were higher than rates reported from France, Taiwan, and Korea [60–62]. In contrast, resistance is alarmingly high in gram-negative bacteria: ciprofloxacin-resistant *E. coli* ranges from 45 to 55% [63, 64], while levofloxacin-resistant *E. coli* reaches 64% [65]. Additionally, the frequency of fluoroquinolone-resistant *Campylobacter* species varied from 0 to 87.3% [66]. A systematic review on ASP implementation in the intensive care unit (ICU) settings reported reductions in hospitalization duration and antimicrobial costs [67]. Our study did not include ICU-specific data. However, it confirmed the reduction in the cost of the antimicrobial agents and supported the hypothesis that the implementation of antimicrobial stewardship programs is beneficial and cost-effective [68].

This study had some limitations. First, it did not include all available antimicrobials because some antimicrobial were not among the restricted and alternative groups defined by the NASP. Second, we could not examine the infectious syndromes of patients and the patterns of antimicrobial resistance. Moreover, although DDD is a recommended metric for antimicrobial consumption by the WHO, it is limited by some factors, which may require considering the use of Days of Therapy/1000 Patient Days instead. This study only addressed the quantity of antimicrobial consumption, while quality control of the antimicrobials used is also an important aspect of a stewardship program. While this study demonstrated significant reductions in antimicrobial consumption post-NASP implementation, we acknowledge that outcomes specific to the Antimicrobial Time-out (ATO) component, such as de-escalation rates or clinician adherence, were not systematically measured. Future evaluations should integrate process metrics to better delineate the ATO’s contribution to stewardship success.

## Conclusion

Our findings illustrated that NASP implementation resulted in reduced use of antimicrobials, especially imipenem, linezolid, vancomycin, and even antifungals, such as caspofungin and amphotericin B liposomal. Moreover, the implementation of this national program reduced the cost of antimicrobial consumption without increasing the length of stay or mortality rate. The implementation of NASP can be beneficial for both medical centers and patients.

## Data Availability

The data that support the findings of this study are available from the corresponding author upon reasonable request.

## Abbreviations

ACI: Antimicrobial consumption index
AMR: Anti-microbial resistance
ASP: Antimicrobial stewardship programs
DDD: Defined daily dose
ESBL-PE: Extended spectrum beta-lactamase producing Enterobacterales
ICU: Intensive care unit
MDR: Multi-drug resistant
NASP: National antimicrobial stewardship program
PTZ: Piperacillin- tazobactam
WHO: World Health Organization
